# Non-Coding RNAs Modulating Estrogen Signaling and Response to Endocrine Therapy in Breast Cancer

**DOI:** 10.3390/cancers15061632

**Published:** 2023-03-07

**Authors:** Oliver Treeck, Silke Haerteis, Olaf Ortmann

**Affiliations:** 1 Department of Gynecology and Obstetrics, University Medical Center Regensburg, 93053 Regensburg, Germany; 2 Institute for Molecular and Cellular Anatomy, University of Regensburg, 93053 Regensburg, Germany

**Keywords:** non-coding RNA, breast cancer, ET, resistance, miRNA, lncRNA, circRNA, tamoxifen

## Abstract

**Simple Summary:**

Endocrine therapy is a common treatment for estrogen-responsive breast cancer, which is used to block the tumor growth-promoting effect of this hormone. The resistance of breast cancer patients to endocrine therapy is a major clinical problem which could be solved by a deeper understanding of its mechanisms. Analysis of the human genome has revealed that messenger RNA (mRNA) coding for proteins represents only a small portion of human RNA. The vast majority of RNAs do not code for proteins and thus are called non-coding RNAs (ncRNAs), which have important functions in health and disease. Here, we summarize current insights into the effects of ncRNAs on estrogen actions in breast cancer cells and their role in endocrine resistance. NcRNAs have been identified which affect the estrogen response and reduce the efficacy of this therapy. Thus, these could be used to predict therapy response and might represent novel therapy targets to overcome endocrine resistance.

**Abstract:**

The largest part of human DNA is transcribed into RNA that does not code for proteins. These non-coding RNAs (ncRNAs) are key regulators of protein-coding gene expression and have been shown to play important roles in health, disease and therapy response. Today, endocrine therapy of ERα-positive breast cancer (BC) is a successful treatment approach, but resistance to this therapy is a major clinical problem. Therefore, a deeper understanding of resistance mechanisms is important to overcome this resistance. An increasing amount of evidence demonstrate that ncRNAs affect the response to endocrine therapy. Thus, ncRNAs are considered versatile biomarkers to predict or monitor therapy response. In this review article, we intend to give a summary and update on the effects of microRNAs (miRNAs), long non-coding RNAs (lncRNAs) and circular RNAs (circRNAs) on estrogen signaling in BC cells, this pathway being the target of endocrine therapy, and their role in therapy resistance. For this purpose, we reviewed articles on these topics listed in the PubMed database. Finally, we provide an assessment regarding the clinical use of these ncRNA types, particularly their circulating forms, as predictive BC biomarkers and their potential role as therapy targets to overcome endocrine resistance.

## 1. Introduction

Estrogens play an important role in the physiology and function of the breast as well as in breast cancer (BC) progression [1]. Their action is mediated by binding to estrogen receptors (ER), namely, the estrogen-activated transcription factors ERα and ERβ and the transmembrane G-protein coupled estrogen receptor (GPER1) [2,3]. Today, ERα is an established target in BC therapy, since estrogen binding to this receptor was recognized early to activate tumor-promoting signaling pathways in BC cells [4,5]. Endocrine therapy (ET) targeting estrogen signaling, primarily with selective estrogen receptor modulators (SERMs) such as tamoxifen and/or aromatase inhibitors (AI) blocking E2 biosynthesis or with the selective estrogen receptor downregulator (SERD) fulvestrant, is currently a standard treatment for patients with ERα-positive BC [6]. However, due to intrinsic or acquired resistance against ET, about 40% of patients relapse after ET and approximately 50% of patients with locally advanced or metastatic ERα-positive BC do not respond to first-line endocrine treatment [7,8]. So far, several mechanisms have been identified underlying intrinsic or de novo endocrine resistance.

Endocrine resistance can emerge from different key mechanisms [9,10]. It can result from impaired estrogen signaling resulting from downregulation, mutation, aberrant phosphorylation, or altered stability of ERα, from an imbalance between ER coactivator and corepressor proteins or ER-binding transcription factors, and from ligand-independent activation of estrogen receptors [11,12,13,14]. Other mechanisms of endocrine resistance include *MYC* overexpression, amplification of cyclin D1 (*CCND1*) or *MDM2* gene, reduced activity of CYP2D6, the enzyme responsible for activation of tamoxifen, aberrant promoter methylation which, e.g., leads to activation of the Akt/mTOR pathway, and activation of other estrogen-independent growth-promoting pathways triggered by mechanisms such as *ERBB2* amplification, bypassing the action of endocrine therapies [8,9,15,16,17,18].

In this review article, we address endocrine resistance mechanisms in ERα-positive BC that are based on modulation of estrogen signaling by non-coding RNAs (ncRNAs). Since ncRNAs have been recognized as an important part of the human transcriptome, in recent years, an increasing number of studies have reported ncRNAs modulating estrogen signaling in BC, and a part of these studies also examined their effect on the response to endocrine therapies in BC patients. In this review, we summarize recent findings and aim to provide an update on the interactions of ncRNAs with estrogen signaling and their implications for ET resistance in ERα-positive BC patients.

## 2. Non-Coding RNAs

Only 2–3% of the mammalian genome codes for messenger RNA (mRNA) that will be translated into proteins. Much of the non-protein coding part of the human genome has historically been regarded as junk DNA. Over the last two decades, however, the development of high-throughput technologies, such as next-generation sequencing, has revealed that a large portion of these genomic regions is transcribed into non-coding RNAs (ncRNAs), which have been shown to regulate diverse cellular functions and pathological processes via gene regulation at all levels of gene expression [19].

Currently, ncRNAs are divided into the subclasses of microRNA (miRNA), long non-coding RNA (lncRNA), circular RNA (circRNA), small nucleolar RNA (snoRNA), Piwi-interacting RNA (piRNA), small nuclear RNA (snRNA), small interfering RNA (siRNA) and the long-known transfer RNA (tRNA) and ribosomal RNA (rRNA) [20]. In this review article, we focus on the role of three regulatory ncRNAs—miRNAs, lncRNAs and circRNAs—in estrogen signaling in BC and their implications for endocrine therapies for this cancer entity. Although there are studies suggesting that some piRNAs and snoRNAs play a role in BC, so far no or insufficient data on interactions of these ncRNAs with estrogen signaling or response to endocrine therapies have been reported.

### 2.1. MicroRNA (miRNA)

MicroRNAs (miRNAs) are small non-coding RNAs of 17–25 nucleotides that are considered master regulators of gene expression. They are able to regulate the expression of target genes by different mechanisms. MicroRNAs form complex regulatory networks in cell development, differentiation, and homeostasis. Dysregulation of miRNA function is associated with an increasing number of human diseases, including cancer. Primarily, miRNAs exert a gene silencing function mediated by mRNA cleavage or degradation and translational repression of the target mRNA [21]. The first step of miRNA silencing mechanisms is the binding and guiding of Argonaute (AGO) proteins to target sites, usually in the 3′ untranslated region (UTR) of mRNAs. The miRNA-loaded AGO is called the miRNA-induced silencing complex (miRISC), which binds to complementary sequences in target mRNA, called miRNA response elements (MREs) [22]. The degree of MRE complementarity determines whether there is AGO2-dependent cleavage of target mRNA or miRISC-mediated translational inhibition and target mRNA decay [23]. A fully complementary miRNA–MRE interaction can induce AGO2 endonuclease activity, leading to mRNA cleavage [23]. In human cells, the majority of miRNA–MRE interactions seem to be not fully complementary, which leads to the blocking of AGO2-mediated mRNA cleavage [24]. Binding of GW182 proteins, DCP1/2, XRN1 and cytoplasmic deadenylase complexes PAN2–PAN3 and CCR4–NOT to the core miRISC complex enables another mechanism of miRNA action, the deadenylation and decapping-dependent degradation of target mRNA by exonuclease XRN1 [24,25]. Furthermore, binding of the translational repressor and decapping activator protein, DDX6, to the miRISC–CCR4–NOT complex has been demonstrated to be a crucial event to reduce stability and translation of the target mRNA [26]. The core miRISC has also been reported to interact with translation regulators such as GIGYF2, EIF4A1/2 and 4EHP, leading to the repression of target mRNA translation [27]. Both AGO and GW182 proteins also serve as molecular hubs for multiple ribonucleoprotein complexes (RNPs) or other enzymes involved in RNA metabolism [28,29]. However, further studies are needed to further elucidate the mechanisms of miRNAs in gene silencing. On the other hand, complexes consisting of miRNA and RNA-binding proteins such as miRNA-containing ribonucleoprotein (miRNP) have been shown to activate gene expression in a post-transcriptional manner [29,30,31].

Another important function of miRNAs is their ability to regulate gene transcription by epigenetic mechanisms modulating both DNA methylation and histone modifications. miRNAs have been shown to affect DNA methylation by two different mechanisms. First, the epigenetic action of miRNAs is mediated by their capability to target and downregulate expression of DNA methyltransferases (DNMTs), such as DNMT1 and DNMT3a and 3b, which are key enzymes in DNA methylation [32]. Second, miRNAs regulate DNA methylation by targeting methylation-related critical proteins, including methyl CpG binding protein 2 (MeCP2) and methyl-CpG binding domain proteins 2 and 4 (MBD2 and MBD4) [33]. In cancer, hypermethylation of tumor suppressor gene promotors and hypomethylation of oncogene promotors is a frequent event involved in carcinogenesis. On the other hand, miRNA genes are also regulated epigenetically. In cancer, oncogenic miRNAs (oncomiRs), which target tumor suppressor genes, are frequently hypomethylated, while tumor-suppressive miRNAs (TsmiR), which target oncogenes, are often hypermethylated. Histone modifications are another key mechanism of epigenetics. MicroRNAs have been shown to regulate gene expression by modulating histone modification via the targeting of histone acetyltransferases (HATs), histone deacetylases (HDACs), histone methyltransferases (HMTs), histone demethylases, histone kinases and histone phosphatases, as well as by modulating histone desumoylation [34]. On the other hand, expression of miRNAs is also regulated by histone modifications.

Most miRNAs are transcribed from DNA sequences into primary miRNAs (pri-miRNAs), which are then further processed by the endoribonucleases DROSHA and DICER1 in the nucleus and cytoplasm, respectively, into precursor miRNAs (pre-miRNAs) and, finally, mature miRNAs, which can bind to AGO proteins to form the RISC (RNA-induced silencing complex), where one strand is selected to become the mature miRNA [35,36]. In addition, there are also non-canonical miRNA biogenesis pathways leading to the generation of functional miRNAs. These include mirtrons that are generated via pre-mRNA splicing and miRNAs generated from small nucleolar RNA (snoRNA) precursors [37].

Generally, a single miRNA species can regulate the expression of hundreds of target genes to a lesser extent, but mRNAs can be targeted by several miRNAs which then act cooperatively to substantially regulate expression of a specific gene [38]. In addition to cellular miRNAs, circulating miRNAs (c-miRNAs) have been identified, e.g., in blood plasma or serum, which result from the release from damaged cells or by active mechanisms [39]. These miRNAs are transported to target cells via protein binding or by vesicles such as exosomes. Recent evidence indicates that c-miRNAs can regulate target gene expression in very distant target cells, thus behaving in a hormone-like way, regulating several key cellular processes in recipient cells, thus affecting cellular development, differentiation, proliferation, cell death and metabolism [40,41,42]. Since these circulating miRNAs have proven to be highly stable, studies have followed suggesting that they have a potential diagnostic, prognostic or predictive power, including in BC [43]. Dysregulation of miRNAs has been increasingly recognized as a critical contributor to cancer development, progression, and therapy resistance. In ERα-positive BC, estrogens binding to ERα trigger a specific transcriptome pattern involving both coding and non-coding RNAs. It has become increasingly clear that miRNAs are not only able to target and downregulate ERα but are in turn also regulated by ERα. The same is true for ERβ and GPER1, which also have been reported to regulate miRNA expression. However, in this review article, we decided to focus on the miRNA effects on estrogen signaling due to the large amount of data on this topic. Several studies have identified miRNAs interacting with estrogen signaling in BC, and some of these miRNAs have been reported to be involved in endocrine resistance. MicroRNAs can regulate estrogen signaling by targeting mRNAs of a wide variety of genes.

#### 2.1.1. MiRNAs Targeting ERα and Its Coregulators

Early studies prompted by identification of miRNAs as novel gene regulatory factors initially identified the *ESR1* gene coding for ERα to be a direct miRNA target. The first miRNA identified to bind to and downregulate *ESR1* mRNA in the context of BC was miRNA-206, which targets the 3′-UTR of *ESR1* mRNA [44]. MiR-206 was also reported to be downregulated in ERα-positive BC [45]. In later studies, *ESR1* mRNA has been shown to be a direct target of a multitude of miRNAs, resulting in its degradation or translational repression [44,46]. A comprehensive study screening the effects of various miRNAs, which were predicted to bind *ESR1* mRNA by in silico analyses, identified miR-22 as exerting the strongest effects on *ESR1* expression by targeting the 3′UTR of its mRNA and thereby inhibiting E2-induced proliferation of MCF-7 cells. MiR-22 has been also shown to be negatively correlated with ERα status [47]. A parallel study performing high-throughput transfections into BC cells of 319 miRNAs predicted to target *ESR1* mRNA identified 21 miRNAs downregulating *ESR1* expression. Among them, miR-206 (corroborating the findings of Adams et al.), miR-18a, miR-18b, miR-193b and miR-302c were confirmed to directly target the 3′-UTR of the *ESR1* gene [48]. *ESR1* silencing decreased expression of ERα-response genes and reduced E2-driven growth. MiR-18a and miR-18b were shown to have a lower expression in ERα-positive tumors, suggesting that these miRNAs have important ERα regulatory functions in BC patients.

Further studies revealed that miRNAs can target and downregulate ERα coregulators (Table 1). MiRNAs have been identified (e.g., miR-9, miR-20a/b, miR-193b and miR-206) that target ER nuclear receptor coactivators NCOA1 and NCOA3, thereby decreasing the cellular estrogen response [48]. Direct binding of the coactivator proteins CBP/p300 to NCOA1 in a complex with ER and DNA promotes histone acetylase activity (HAT), allowing expression of ER-responsive genes [49]. The miRNAs miR-766-5p and miR-4474/4717 have been identified to directly target the *CREBBP* gene, leading to downregulation of the estrogen effector gene *MYC* [50,51]. Regarding ER corepressors, the *NCOR1* gene has been proven to be targeted by miR-1587 [52], and the *NCOR2* gene was shown to be targeted by miR-10a/b miR-100-5p [53,54], with the downregulation of both corepressors expected to enhance estrogen signaling. The orphan nuclear receptor NR5A2 (nuclear receptor subfamily 5, group A, member 2) cooperates with ERα in the activation of ERE-dependent genes [55]. MiRNAs such as miR-27b, which target and downregulate NR5A2, are thus expected to significantly affect estrogen signaling [56]. In recent years, it has been recognized that the transcriptional activity of Erα is dependent on the pioneer transcription factors FOXA1 and GATA3, which are master transcriptional regulators and important key players in the estrogen response. Forkhead protein FOXA1 is a key determinant of estrogen signaling and the endocrine response and is necessary both for ER binding to chromatinized DNA and the gene regulatory function of this nuclear receptor [57]. The ER-triggered regulation of 95% of estrogen-responsive genes requires FOXA1. Targeting of FOXA1 expression by the miR-100 and miR-132 miRNAs results in suppression of E2-dependent BC cell growth [58,59]. Like FOXA1, GATA binding protein 3, GATA3, also acts as a pioneer transcription factor in estrogen signaling by the formation of chromatin loops which allow the subsequent recruitment of ERα complexes regulating gene expression [60]. The *GATA3* mRNA has been reported to be targeted and downregulated by the miRNAs miR-155, miR-206 and miR-433-3p [48,61,62]. The transcription factors specificity protein 1 (SP1), activating-protein 1 (AP1), NFkB and RUNX3 are known to directly bind to ERα and ERβ, with these dimers enabling a broadened estrogen response in a context-specific manner by binding to alternative response elements and thereby activating a distinct set of genes, including the well-known estrogen target genes *PGR* and *MYC*. Thus, miRNAs targeting the expression of the *SP1* gene or the *JUN* or *FOS* genes, which code for the proteins assembling to AP1, are also important modulators of estrogen signaling. MiR-22 is an example of a miRNA that targets and downregulates *SP1* gene expression [63]. The Forkhead transcription factor FOXM1 is another important protein interacting with ERα to regulate gene expression [64]. MiR-23 has been reported to be inversely correlated with FOXM1 in BC, and further studies confirmed the direct targeting of this transcription factor by miR-23, resulting in downregulation of MYC and cyclin D1 and growth inhibition [65,66]. Cyclin D1 coded by *CCND1* gene is not only an estrogen-responsive effector and mediator of proliferative estrogen actions, but it itself binds to ERα and its coactivators NCOA1 and NCOA3, thus upregulating the transcriptional activity of ERα [67]. Several miRNAs targeting and downregulating expression of *CCND1* have been identified, among them miR-15a/b, miR-17-5p, miR-20a-5p, miR-193b-3p and miR-206, the latter of which has been demonstrated to reduce estrogen-triggered proliferation [68].

A recent study with the goal to identify the miRNA most highly upregulated upon E2 treatment in BC cells performed a high-throughput sequencing of paired mRNA and miRNA expression, resulting in the identification of miR-503 as a master regulator of the estrogen response in MCF-7 cells [69]. In this study, miR-503 was found to be the most estrogen-responsive miRNA as well as the one with the largest impact on E2 responsive gene expression. MiR-503 targeted and reduced mRNA levels of *CCND1*, known to be induced by ERα and an important mediator of estrogen-triggered proliferation; this, in turn, stimulated the transcriptional activity of ERα via NCOA1 [70,71]. Notably, it was also shown that estrogen-induced miR-503 degraded *ZNF217* mRNA by targeting its 3′-UTR. The *ZNF217* gene, coding for zinc finger protein 127, is a transcriptional regulator and oncogene in BC with an important role in the estrogen response [72]. ZNF217 binds to ERα, thereby increasing the recruitment of ERα to estrogen response elements (EREs), resulting in amplification of the estrogen response in BC [73]. Notably, this miR-503 target has not only been identified as a biomarker of poor survival in patients with Luminal A breast tumors [74], but low levels of ZNF217 in pre-treatment tumor samples, which result from an E2-triggered increase of miR-503 expression, were associated with a good response to neoadjuvant ET and were also shown to be associated with longer relapse-free survival (RFS) in patients treated with ET only [75]. Since miR-503 is strongly induced by ERα activation, but targets and downregulates several genes mediating the proliferative ERα response, it is considered to be part of a negative feedback loop limiting the dysregulated E2-driven proliferation of BC cells. Thus, miR-503 might be a promising molecule for treatment of ERα-positive BC.

**Table 1:** MiRNAs directly targeting the mRNA of *ESR1,* or other genes involved in estrogen signaling or the estrogen response, and their in vitro effect on sensitivity of BC cells to ET drugs. To date, not every miRNA–mRNA interaction shown here has been examined with regard to ET drug response.

miRNA	Target	Cellular Response to ET Drugs	Reference
let-7	*ESR1, CYP19A1*		[48,76]
let-7b, 7i	*ERα36*	Tamoxifen sensitivity ↑	[77,78]
miR-7-5p	*FOS, EGFR*	Tamoxifen resistance ↑	[79]
miR-9-5p	*ESR1, NCOA3*	Tamoxifen resistance ↑	[48,80,81,82]
miR-10b	*NCOR2*		[53]
miR-15b	*FOXO1, CCND1*	Tamoxifen resistance ↑	[83,84,85,86]
miR-17-5p	*NCOA3, CCND1*		[87]
miR-18a	*ESR1*	Tamoxifen resistance ↑	[48,88,89,90,91]
miR-19a/b	*ESR1, CYP19A1, CCND1*		[88,92]
miR-20a/b	*ESR1, NCOA3, CCND1*		[88]
miR-21	*ESR1, PTEN*	Tamoxifen + Fulvestrant resistance ↑ (AI ?)	[93,94,95,96,97,98]
miR-22	*ESR1, SP1*		[47,81,99]
miR-26a/b	*ESR1*		[100,101]
miR-27b	*NR5A2, CREB1*	Tamoxifen sensitivity ↑	[56]
miR-30a-5p	*FOXA1*		[102]
miR-34a	*CCND1*		[103]
miR-92a-3p	*ESR2*		[104]
miR-100	*FOXA1, NCOR2*		[54,58]
miR-129	*ESR1, ESR2*		[105]
miR-130b	*ESR1, CCNG2*	Tamoxifen resistance ↑	[106,107,108]
miR-132	*FOXA1*		[59]
miR-135a	*ESR1, ESRRA, NCOA1*	Tamoxifen sensitivity ↑	[109]
miR-142	*ESR1*	Tamoxifen resistance ↑	[110]
miR-145-5p	*ESR1*		[111]
miR-155	*GATA3, CEBPB*	AI resistance ↑ Tamoxifen resistance ↑	[112,113,114,115]
miR-181a	*PGR, ESR1*	Tamoxifen resistance ↑	[116,117]
miR-192-5p	*ESR1*	Tamoxifen resistance ↑	[118]
miR-193b	*ESR1, NCOA3, CCND1*		[48,80]
miR-205	*MED1*	Tamoxifen resistance ↑ AI resistance ↑	[119,120,121,122]
miR-206	*ESR1, NCOA1, NCOA3, GATA3, CCND1*		[44,45,48,68,123]
miR-219b	*ESR1*		[48]
miR-221	*ESR1, CDKN1B*	Tamoxifen + Fulvestrant resistance ↑	[48,124,125,126,127,128]
miR-222	*ESR1, CDKN1B*	Tamoxifen + Fulvestrant resistance ↑	[48,124,125,126,127,128]
miR-302	*ESR1, CCND1*		[48]
miR-320a	*ESRRG*	Tamoxifen sensitivity ↑	[129]
miR-335	*ESR1*	Tamoxifen resistance ↑	[130]
miR-342-3p	*ID4*	Tamoxifen sensitivity ↑	[131,132,133,134]
miR-373	*ESR1*		[135]
miR-378a-3p	*PGR*		[116]
miR-424	*ESR1, GPER1, CCND1*	AI sensitivity ↑	[122,136,137,138]
miR-433-3p	*GATA3*		[61]
miR-449a	*CCND1, ADAM22*	Tamoxifen sensitivity ↑	[139]
miR-451	*ESR1, MYC*	Tamoxifen sensitivity ↑	[140,141]
miR-484	*KLF4*	Tamoxifen sensitivity ↑	[142,143]
miR-489	*MAPK11*	Tamoxifen sensitivity ↑	[144]
miR-520a-3p	*ESR1, CCND1*		[145]
miR-520d-3p	*ESR1*	Fulvestrant resistance ↑	[146]
miR-874	*ESR1*		[147]
miR-1587	*NCOR1*		[52]
miR-4728-3p	*ESR1*		[148,149]

#### 2.1.2. MiRNAs Targeting ERβ, GPER1 and Estrogen Effector Genes

Since the function of ERβ encoded by the *ESR2* gene in BC has, for a long time, not been convincingly described due to inconsistent studies mainly based on the use of unspecific antibodies, few studies have been performed validating the *ESR2* gene as an miRNA target, e.g., of miR-92 or miR-30a [104,150], although databases such as TargetScan [151] or miRTarBase [152] predict it to be targeted by a variety of miRNAs. Although the same is true for the third estrogen receptor GPER1, the effect of miRNAs on this gene has also been addressed in few studies, which report that miR-424 downregulates and miR-399 upregulates GPER1 [138,153]. MiR-19a-3p is an example of a miRNA targeting an important enzyme of steroidogenesis, the aromatase CYP19A1, which catalyzes the conversion of androgens into estrogens [154]. It would be not surprising if miRNAs downregulating this important enzyme, leading to decreased estrogen levels, would trigger resistance to aromatase inhibitor therapy. The nuclear estrogen-related receptors (ERRs) α, β and γ do not bind estrogens, but interfere with estrogen signaling by different mechanisms [155]. ERRα (ESRRA) mRNA is targeted by miR-137, which has been reported to suppress BC cell proliferation, and by miR-497 [156,157].

Finally, miRNAs can regulate expression of estrogen response downstream effector genes such as *MYC*, which is induced by enhancer binding of ERα and AP1 and is involved in the regulation of about 15% of human genes [158,159]. *MYC* is often upregulated in BC, activating the expression of many proliferation-promoting genes such as cyclins but repressing expression of p21^Waf1^ (*CDKNA1*) [160,161,162]. Consequently, miRNAs targeting *MYC* have major effects. For example, miR-34c direct targeting of *MYC* mRNA has been reported to decrease MYC-dependent DNA replication [163]. As discussed above, the *CCND1* gene, coding for cyclin D1, acts both as an ERα coactivator and as an important estrogen effector gene mediating the proliferative effect of estrogens. Thus, miRNAs such as miR-206 which target the *CCND1* mRNA have been shown to suppress BC cell proliferation [68]. MiR-15a and miR-16 have been reported to promote apoptosis by targeting another important target gene of estrogen signaling, the apoptosis inhibitor gene *BCL2*, which is activated via two estrogen-responsive elements located within its coding region [164,165].

#### 2.1.3. Circulating miRNAs Affecting Estrogen Signaling in BC

Cells, including tumor cells, release miRNAs into the microenvironment or the bloodstream to regulate target cell gene expression in a paracrine or endocrine manner. Two alternative mechanisms protect secreted miRNAs from RNase degradation. First, free circulating miRNAs (c-miRNAs) can form stabilizing complexes with the argonaute protein (AGO) and other RNA-binding proteins or with high- and low-density lipoproteins [166]. The second mechanism of protection of secreted miRNAs is their transport as cargo of extracellular vesicles (EV) such as exosomes. Exosomes are a major subtype of extracellular vesicles that allow endocrine crosstalk between distant organs and are involved in disease development. With regard to cancer, circulating exosomes can transport various biomolecules, including tumor promotor and suppressor proteins, to target tissues [167,168]. Exosomes with a highly specific miRNA cargo (exomiRs) are released by tumor cells into different body fluids, including the bloodstream [169]. Circulating free miRNAs and exomiRs have been proposed as promising non-invasive biomarkers for cancer diagnosis, prognosis, treatment and monitoring of disease progression [170]. Currently, the potential of exomiRs is being assessed as a novel therapy strategy of cancer and other diseases [171,172,173]. In BC, c-miRNAs and exomiRs have been shown to affect essential features such as tumor growth, invasion and angiogenesis [174]. For example, the circulating exosomal miRNAs miR-92 and miR-25-3p in BC patients can induce angiogenesis and epithelial–mesenchymal transition (EMT). ExomiRs have also been reported to affect estrogen signaling in BC cells. However, it has to be considered that miRNAs always target various genes, and the reported effect on BC was not always proven to result from targeting the estrogen response. With regard to the intrinsic molecular subtypes of BC, the Luminal A type, which is the most estrogen-responsive one, was reported to exhibit higher levels of the exomiRs miR-142-5p and miR-320a, which might be interpreted as a compensatory mechanism to impair the estrogen response since these miRNAs are known to target *ESR1* and *ESRRG* [175] (Table 1). Levels of exomiR-222, known to target the *ESR1* and *CDKN1B* genes, were reported to be decreased in Luminal A BC and to distinguish this type from the basal-like and Luminal B-intrinsic subtype [176]. Exosomal miR-21, which is known to target and downregulate *ESR1* gene expression, was found to be elevated in ERα-negative BC patients and to promote invasiveness of BC cells [177]. The same effect has been reported for exomiR-10b targeting *NCOR3* [178]. Exosomal miR-373, which is known to target and downregulate *ESR1* gene expression, has also been found to be elevated in ERα-negative BC patients and to highly correlate with BC invasion and migration [135].

#### 2.1.4. Effect of miRNAs Targeting Genes of Estrogen Signaling on Response to Endocrine Therapies

As shown above, miRNAs can affect estrogen signaling at all levels, from steroidogenic enzymes to estrogen receptors and from various co-regulatory proteins to estrogen response genes, the targets of estrogen signaling. The decreased expression of various genes involved in cellular estrogen signaling resulting from specific miRNA–mRNA interactions has been shown to affect endocrine resistance. Although miRNA-triggered downregulation of ERα expression can result in a decrease of its immunohistochemistry score, it does not totally suppress ERα levels, thus usually sustaining the “ER-positive” status of the respective BC tissue, allowing the use of ET regimens. However, downregulation of ERα expression by miRNAs has been shown to diminish the efficacy of endocrine therapies. MiRNAs have also been reported to target other genes involved in estrogen signaling or the estrogen response and to thereby affect the response to ET (Table 1).

##### MiRNAs and Tamoxifen Resistance

The regulation of various genes involved in estrogen signaling by miRNAs has been reported to affect the response to tamoxifen therapy. With regard to estrogen signaling, the *ESR1* gene has been very thoroughly examined to identify miRNAs directly targeting its mRNA. Downregulation of *ESR1* expression by miRNAs is considered to be an important mechanism for miRNA-triggered resistance to tamoxifen. To date, a variety of miRNAs have been identified that target *ESR1* and thereby enhance tamoxifen resistance. However, an impaired tamoxifen response has also been shown to result from the interaction of miRNAs with other mRNAs of genes involved in estrogen signaling (Table 1). Among the miRNAs increasing tamoxifen resistance are miR-9 (targeting *ESR1* and *NCOA3*), miR-15b (via *FOXO1* and *CCND1*), miR-18a (*ESR1*), miR-21 (*ESR1*), miR-130b (via *ESR1* and *CCNG2*), miR-142-5p and miR-320a (via *ESR1* and *ESRRG*), miR-155 (via *GATA3* and *CEBPB*), miR-192-5p (*ESR1*), miR-205 (targeting *MED1*), miR-221/222 (*ESR1*) and miR-355 (*ESR1*) (Table 1). Interestingly, some of these miRNAs can act in a paracrine or endocrine manner when bound to specific proteins or as cargo of exosomes. Exosomes secreted from tamoxifen-resistant tumors are reported to transmit resistance partly via delivery of miR-9-5p, which targets *ESR1* and *NCOA3* [82]. Exosomal miR-221/222 has been reported to confer tamoxifen resistance to recipient ERα-positive BC cells via downregulation of this receptor [125].

On the other hand, miRNAs have been identified which enhance the tamoxifen response by different mechanisms. MiR-15a and miR-16 downregulate expression of the ERα target gene *BCL2*, thereby promoting tamoxifen sensitivity by reducing the action of anti-apoptotic BCL2 [165]. Downregulation of ERα-interacting transcription factors AP-1, Sp-1, NFκB and the forkhead transcription factor FOXM1 was associated with enhanced endocrine sensitivity [179,180]. Thus, miRNAs targeting one of these genes, such as miR-22 or miR-23, were suggested to counteract antiestrogen resistance. MiR-100 and miR-132 targeting of *FOXA1* mRNA has been reported to increase tamoxifen sensitivity since FOXA1 is necessary for tamoxifen-bound ER recruitment to chromatin, resulting in the repression of estrogen-response genes [57]. Overexpression of miR-342 was shown to sensitize MCF-7 BC cells to tamoxifen actions such as apoptosis induction and inhibition of proliferation by targeting inhibitor of differentiation 4 (ID4), a regulator of estrogen signaling [132,181]. Furthermore, miR-449 was reported to enhance tamoxifen sensitivity by targeting ADAM22 (ADAM metallopeptidase domain 22), which is regulated by the ER coactivator NCOA3, and which was reported to be an ER-independent mediator of tamoxifen resistance [182]. MiR-27b increases tamoxifen sensitivity by downregulation of the transcription factor NR5A2, which cooperates with ERα and ERβ in the regulation of *CYP19A1* gene expression [183]. MiR-320a has been reported to promote tamoxifen sensitivity by targeting ERRγ (*ESRRG*) mRNA [129,184]. A very recent study aimed to elucidate the role of miR-489 in endocrine resistance since this miRNA has been found to be downregulated in tamoxifen-resistant BC [185,186]. In this study, expression of miR-489, which has previously been suggested to act as a tumor suppressor in different cancer entities, including BC, and to exert anti-proliferative actions on BC cell lines in vitro [187], was upregulated by E2 and reduced after E2 deprivation in ERα-positive cell lines. Additionally, its expression was considerably downregulated in two different tamoxifen-resistant MCF-7 cell lines, but miR-489 overexpression restored tamoxifen sensitivity. Further analyses demonstrated that miR-489 downregulated *TFF1* and *PGR* mRNAs, suggesting its function as a negative regulator of estrogen signaling. The association of low miR-489 expression in ERα-positive BC with worse overall survival further supported an essential tumor-suppressive role of E2-induced miR-489 in this cancer entity, which is suggested to be at least partially mediated by a negative feedback loop between miR-489 and estrogen signaling. Considering the ability of miR-489 overexpression to restore tamoxifen sensitivity in vitro, the authors concluded that miR-489 might be an interesting treatment option for patients with tamoxifen-resistant BC [144].

##### MiRNAs and Response to Fulvestrant and Aromatase Inhibitors

The response of ERα-positive BC patients to the SERD fulvestrant is affected by various mechanisms. Downregulation of *ESR1* gene expression by miRNAs has been shown to be one mechanism of fulvestrant resistance. A recent study examining predictors of fulvestrant long-term benefits reported the *ESR1*-targeting miR-520d-3p to be negatively associated with 18-month progression-free survival (PFS) [48,146].

Another miRNA reported to target *ESR1* mRNA, miR-21, has been demonstrated to confer resistance both to fulvestrant and tamoxifen [93]. MiR-221/222, which also target and downregulate *ESR1* gene expression, have been shown to confer acquired fulvestrant resistance [127,128]. More recently, mRNA expression profiling of fulvestrant-resistant MCF-7 cells subjected to miR-221/222 knockdown revealed the upregulation of 428 genes and downregulation of 224 genes, highlighting the complex functional role of miR-221/222 in fulvestrant resistance and uncovering further pathways affected by these miRNAs that are involved in the fulvestrant response, such as Wnt/β-catenin, EGFR, TGFβ, Notch, Jak–STAT, ERBB2, MAPK and p53 [128]. A number of other miRNAs have been observed to be dysregulated in fulvestrant-resistant BC cells; however, their effect on the response to this antiestrogen still has to be examined [188].

The use of aromatase inhibitors, such as letrozole and anastrozole, is another important ET regimen in postmenopausal ERα-positive BC patients. However, the development of acquired AI resistance is a critical clinical challenge. Emerging studies have determined that, like tamoxifen and fulvestrant resistance, miRNAs can also mediate AI resistance. Although the number of studies examining this connection is still limited, it has been shown that miRNAs downregulating the expression of CYP19A1 aromatase, which catalyzes the conversion of androgens into estrogens, are able to confer AI resistance. MiR-19a-3p has been reported to downregulate this important enzyme of steroidogenesis by targeting the *CYP19A1* mRNA, resulting in decreased AI sensitivity [154]. In another study, high baseline miR-155 expression was found to correlate with poor response to AI therapy in a cohort of ERα-positive BC patients treated with the neoadjuvant anastrozole. The authors suggested that miR-155 might represent a biomarker potentially capable of predicting the clinical response to AI therapy [113]. Although this miRNA was not reported to downregulate aromatase expression, it is known to target GATA3, which acts as a pioneer transcription factor in estrogen signaling by formation of chromatin loops, allowing binding of ERα to its target genes [60].

### 2.2. Long Non-Coding RNA (lncRNA)

Among the non-protein coding transcripts, a class referred to as long non-coding RNA (lncRNA) has attracted increasing attention. LncRNAs are non-coding transcripts longer than 200 nucleotides comprising a heterogeneous class of sense or antisense transcripts that overlap other genes, intronic lncRNAs, enhancer RNAs (eRNAs) and the large intergenic non-coding RNAs (lincRNAs) [189]. Genetically, lncRNAs can be broadly classified into those regulating local chromatin structure and/or gene expression in cis versus those that leave the site of transcription and perform regulatory functions in trans [190].

LncRNAs regulate gene expression at different levels. First, lncRNAs can regulate chromatin condensation and histone modification by different mechanisms, acting as a signal for chromatin remodeling proteins, as a scaffold for ribonuclear protein assembly or as a signal for transcription factors. LncRNAs were also suggested to regulate epigenetic silencing by altering DNA methylation patterns [191].

Second, they can regulate transcription by guiding transcription factors to gene promotors by binding to RNA polymerase II and by directly affecting mRNA splicing [192]. Furthermore, they can act post-transcriptionally, as a decoy preventing target binding of proteins, or by reducing mRNA stability and interacting with miRNA. Regarding lncRNA–miRNA interactions, lncRNAs can function as endogenous competitor RNAs (ceRNAs) by acting as miRNA sponges, presenting binding sequences for miRNAs, which results in reduced miRNA availability and impaired miRNA–mRNA binding.

As an endogenous competitor RNA (ceRNA), long non-coding RNA can also directly compete with miRNAs for the target site of mRNA. Finally, lncRNA is known to bind to various RNA-binding proteins (RBPs), thereby affecting the stability of either the lncRNA or RBP, which regulates RNA metabolism, including N6-methyladenosine (m6A) modification and alternative splicing as well as affecting subcellular localization [193]. Furthermore, lncRNAs can act at the post-translational level by recruiting translational repressors [194,195,196]. It has also been shown that lncRNAs can bind to key signaling proteins, affecting, e.g., their phosphorylation, thus directly regulating their signaling pathways [197]. Recent studies demonstrated that some transcripts that are annotated as lncRNAs actually encode for small proteins and that lncRNAs can be processed into small ncRNAs such as miRNAs or piRNAs [198,199,200]. LncRNAs can exert oncogenic or tumor suppressor functions affecting different biological processes such as cell proliferation, cell death and drug resistance [201,202,203]. LncRNAs can be secreted into the circulation system via exosomes or bound to RBPs. Circulating lncRNAs are considered promising diagnostic biomarkers for various diseases, including cancer [204].

#### 2.2.1. Long Non-Coding RNAs Affecting Genes Involved in Estrogen Signaling and Response to Endocrine Therapies

LncRNAs have been demonstrated to affect the response to endocrine therapies (ET) by different mechanisms [205]. The regulation of components of estrogen signaling pathways is one important mechanism, which is, inter alia, mediated by the function of lncRNAs as ceRNA blocking miRNA action. With regard to the response to ET, lncRNAs are able to both increase the ET response by maintaining expression of its target genes and to diminish the ET response by increasing constitutive estrogen signaling. In comparison with miRNAs, to date, fewer studies have been published addressing the role of lncRNAs in this context.

##### LncRNAs Affecting Response to Tamoxifen or Fulvestrant via Modulation of Estrogen Signaling

In the last few years, an increasing amount of evidence has demonstrated a role for lncRNAs in estrogen signaling and therapy response. A recent study reported that the lncRNA ERLC1 (ERα-regulated long noncoding RNA 1) counteracted ERα downregulation by miR-129 and tethered FXR1, resulting in a positive feedback loop that potentiated ERα signaling which, in turn, led to cellular resistance to tamoxifen and fulvestrant [105]. Another recent study reported that lncRNA LINP1 downregulated ERα expression, and although the mechanisms of this interaction remain to be elucidated, this lncRNA was shown to confer tamoxifen resistance to BC cells. LINP1 was increased in tamoxifen-resistant BC cells, and LINP1 knockdown significantly increased tamoxifen-triggered apoptosis and reduced tamoxifen resistance in vitro and in vivo. Mechanistically, LINP1 was reported to be a direct target of ER-mediated transcriptional repression, resulting in increased LINP1 expression upon tamoxifen treatment. Thus, the authors of this study suggested this lncRNA as a putative target to improve tamoxifen sensitivity [206]. As mentioned above, the important oncoprotein cyclin D1 (*CCND1*) is not only an ERα target and mediator of proliferative estrogen actions, but it itself binds to Erα, enhancing its transcriptional activity. Furthermore, amplification of the *CCND1* gene and overexpression of the cyclin D1 protein have been shown to be associated with tamoxifen resistance. A recently identified lncRNA, DILA1, was shown to directly bind to the cyclin D1 protein, thereby inhibiting its phosphorylation at threonine-286, leading to reduced ubiquitination and degradation and thus to increased stability of cyclin D1. This function of DILA1 resulted in the overexpression of cyclin D1 in BC cells and thus contributed to tamoxifen resistance. DILA1 knockdown decreased BC cell proliferation and was able to restore tamoxifen sensitivity both in vitro and in vivo. Furthermore, high expression of DILA1 in BC tissue was associated both with cyclin D1 overexpression and with poor prognosis in BC patients who received tamoxifen treatment, suggesting that DILA1 might be an interesting therapeutic target to downregulate cyclin D1 and reverse tamoxifen resistance in BC patients [197]. HOX transcript antisense RNA (HOTAIR), which participates in EMT and the maintenance of BC stem cells, has been identified as the first lncRNA correlated to poor prognosis for BC [207]. HOTAIR was demonstrated to elevate ERα occupancy on chromatin, which results in the overexpression of estrogen response genes and contributes to ET resistance. As another molecular mechanism of ET resistance, this lncRNA, which is upregulated in tamoxifen-resistant BC, was reported to trigger ligand-independent ERα actions [208]. A recent in silico study suggested the effect of HOTAIR on ET response to be also mediated by blocking the miR-130b-3p-mediated limitation of *ESR1* expression, leading to hyperactive estrogen signaling [106]. H19, the lncRNA most studied with regard to BC, is upregulated in tamoxifen-resistant tumors and affects the ET response via different mechanisms [205]. In resistant cells, silencing of H19 increased the sensitivity to tamoxifen, resulting in a tamoxifen-triggered decrease in cell proliferation, invasion ability, cell survival and increased apoptosis [209]. With regard to estrogen signaling, H19 has been demonstrated to confer resistance to tamoxifen and fulvestrant via the upregulation of ERα expression and by protecting ERα from fulvestrant-triggered protein degradation [210]. LINC-ROR is another lncRNA involved in tamoxifen resistance and was reported to act as a sponge for miR-205-5p. LINC-ROR is positively associated with tamoxifen resistance, and its knockout enhanced BC cell sensitivity to tamoxifen. This effect of LINC-ROR was suggested to result, among other mechanisms, from enabling estrogen-independent ERα signaling by affecting estrogen receptor activation through the MAPK/ERK pathway, specifically by facilitating the degradation of the ERK-phosphatase DUSP7 [211]. DSCAM-AS1 (down syndrome cell adhesion molecule-antisense RNA 1) is a lncRNA containing estrogen response elements (EREs) in its promoter region and has been identified to be the most strongly estrogen-responsive lncRNA in BC [212]. DSCAM-AS1 is upregulated by estrogens and downregulated by tamoxifen. DSCAM-AS1 was reported to be overexpressed in BC tissues and in tamoxifen-resistant cells and to be positively associated with high tumor grade and metastasis [212]. Furthermore, DSCAM-AS1 has been demonstrated to be an independent prognostic factor of poor survival in luminal BC patients treated with ET [213]. Thus, DSCAM-AS1 has been suggested as a good predictive biomarker and possible therapy target for this kind of BC. Mechanistically, DSCAM-AS1 was shown to act as a sponge for miR-137, which is known to prompt cell cycle arrest at the G0/G1 phase, leading to enhanced proliferation and inhibition of apoptosis in tamoxifen-resistant BC cells [214]. DSCAM-AS1 expression is not only highly responsive to estrogens, but its silencing or knockout was, in turn, demonstrated to decrease expression of ERα and its coregulator FOXA1 [215]. Thymopoietin antisense transcript 1 (TMPO-AS1) is a lncRNA reported to positively regulate *ESR1* expression by stabilizing *ESR1* mRNA. In tamoxifen-resistant MCF-7 xenograft models, knockdown of TMPO-AS1 significantly reduced tumor growth, suggesting that this lncRNA might be an interesting therapy target for patients resistant against this SERM [216]. LOL (lncRNA of luminal) is a lncRNA highly expressed particularly in luminal BC. Survival analysis of 374 luminal BC samples indicated that LOL is an independent prognostic factor for poor survival in luminal BC. LOL was reported to act as a sponge for let-7 miRNA, which is known to target and downregulate *ESR1* mRNA. Knockdown of LOL in tamoxifen-resistant MCF-7 cells recovered the sensitivity to tamoxifen, suggesting that LOL contributes to tamoxifen resistance [217]. Metastasis-associated lung adenocarcinoma transcript 1 (MALAT1) is a lncRNA that has been reported to be overexpressed in BC, being associated with poor relapse-free survival (RFS) [218]. High expression of MALAT1 was associated with overexpression of ERα and its targets PR and cyclin D1 and was also shown to reduce the response to tamoxifen, as it is associated with a short RFS in tamoxifen-treated patients [219]. This effect of MALAT1 was suggested to result from its ability to regulate both *ESR1* mRNA splicing and gene expression [220]. Finally, the lncRNA SRA1, which has been identified as binding to the nuclear receptor coactivator NCOA1, acts as an RNA coactivator in this complex, increasing the transcriptional activity of steroid hormone receptors, including ERα [221]. SRA1 expression was found to be elevated in breast tumors compared with adjacent normal breast tissue. SRA1 coactivation of ERα (specifically, its AF-1 domain) has been reported to enhance the agonist activity of tamoxifen-bound ERα and was suggested to contribute to tamoxifen resistance [222]. The expression of SRA1 gene is complex, as it not only codes for lncRNAs of different lengths, but also encodes the protein SRAP (steroid receptor co-activator protein) [223]. The SRAP protein modulates the activity of nuclear receptors such as ERα and is involved in the regulation of splicing and the cell cycle [224]. The lncRNA SRA1 functions as a scaffold, and it also interacts with proteins of miRNA processing (DICER1 and TRBP), with RISC components such as AGO2 and with transcription factors such as FOXO1. FOXO1 affects estrogen signaling by regulating isoform-specific transactivation of the ER target PR and has been reported to be involved in tamoxifen resistance [85,225,226]. Taken together, both SRA1 lncRNA and SRAP protein modulate the activity of ERα, and current data suggest that both are involved in tamoxifen resistance, mediated by the modulation of key genes such as *ESR1*, *PGR* and *FOXO1*.

##### LncRNAs Affecting Estrogen Signaling and Response to Aromatase Inhibitors

In BC treatment, the resistance to aromatase inhibitors (AI) is an ongoing challenge to be urgently solved. LncRNAs have been shown to be involved in AI resistance, among other mechanisms, via regulation of estrogen signaling or metabolism. A recent study identified the intergenic lncRNA LINC00094 as increasing the sensitivity of ERα-positive BC cells to letrozole. LINC00094 was shown to increase and maintain the expression of aromatase (CYP19A1), the enzyme targeted by aromatase inhibitors (AI). The effect of LINC00094 on letrozole response was due to its sponging of miR-19a-3p, a miRNA known to downregulate *CYP19A1* mRNA and to inhibit the EMT process in BC, both promoting letrozole sensitivity [154]. The lncRNA MIR2052HG was reported to increase ERα expression, leading to augmented cell proliferation in ERα+ BC cells and consequently reducing the response to AIs in adjuvant therapy for early-stage BC. However, when BC cells were treated with AIs, a decrease in both MIR2052HG lncRNA and *ESR1* expression was evident. Overexpression of this lncRNA enhanced cell proliferation, colony formation, and ERα expression, corroborating the positive MIR2052HG–ERα association. Moreover, knockdown of lncRNA MIR2052HG led to an increase in p-AKT levels which promoted downregulation of p-FOXO3 and total FOXO3; consequently, *ESR1* expression was downregulated. Thus, the authors proposed MIR2052HG as a potential AI response biomarker [227]. However, more studies are needed to corroborate the effect of lncRNA MIR2052HG on the AI response.

##### Exosomal Transport of lncRNAs

Like miRNAs, lncRNAs can be secreted into vesicles such as exosomes to regulate the tumor microenvironment or, if transported by the bloodstream, to exert endocrine actions such as regulation of angiogenesis and metastatic growth. To date, over 15,000 different lncRNA molecules can be found in the web-based database exoRBase of ncRNAs in human blood exosomes [228]. MALAT1 is a prominent lncRNA found in exosomes from BC patients. MALAT1 is known to activate ERα and its target, the tumor promotor cyclin D1, and is oncogenic in BC via different mechanisms [229]. However, the activities of miRNAs and lncRNAs in cancer cell exosomes have mostly been characterized in in vitro studies; thus, research is needed to validate the role of these ncRNAs in the ET response or metastasis in BC patients.

LncRNAs affecting estrogen signaling or metabolism and the response to ET continue to be discovered via bioinformatic analysis of BC data. As suggested previously, much more research is needed to understand the roles of lncRNAs in BC and in ET resistance, since they are putatively highly potent targets for tumor therapy and for overcoming endocrine resistance.

### 2.3. Circular RNA (circRNA)

CircRNAs are a class of ncRNAs that covalently form a closed loop in eukaryotes. They are transcribed from protein-coding genes and further processed by an unconventional pre-mRNA splicing mechanism, referred to as backsplicing, in which the 3′-end of an exon is ligated to the 5′-end donor splice site of the same or an upstream exon [230]. Based on their structure, derived from different modes of biogenesis, circRNAs can be classified as exonic, exon-intronic, circularized intronic and tRNA intronic, with the latter constituting 85% of all circRNAs. In the process of circRNA biogenesis, the distant donor and acceptor splice sites need to be brought into proximity to be ligated to form a circular molecule, a process which is mediated by different mechanisms [231]. Their circular form makes circRNAs resistant to degradation by exonucleases, which results in their longer half-life and higher copy numbers in cells [232]. Due to their stability, circRNAs have been suggested to be good diagnostic markers in various diseases, including cancer. However, recent studies demonstrated degradation of circRNAs by specific intracellular RNases, establishing cellular circRNA homeostasis, and dysregulation of these processes can lead to cellular aberrations and disease [233]. With regard to cancer, circRNAs can be divided into oncogenic and tumor-suppressive molecules. In normal cells, both types are in a well-balanced ratio, and its dysregulation is thought to contribute to tumorigenesis. The function of circRNAs is mediated by different molecular mechanisms. First, circRNAs can indirectly regulate gene expression by acting as a sponge for miRNAs [234,235]. CircRNAs often contain miRNA response elements (MREs) which can bind miRNAs, preventing them from silencing their target mRNAs. Thus, these circRNAs are classified as competing endogenous RNAs (ceRNAs) [234]. CircRNAs have been reported to contain MREs for several miRNAs, or up to 70 MREs for one specific miRNA [235]. Later, large-scale screening studies showed that many circRNAs have no or only limited numbers of MREs, suggesting further mechanisms of circRNA action. Another mechanism of transcriptional regulation by circRNAs is the modulation of mRNA splicing by intron retention, which can result in truncated proteins or the inhibition of mRNA transport into the cytoplasm. Second, circRNA can bind to RBPs, thereby exerting different actions. On the one hand, circRNA can act as a scaffold for RBP protein complex assembly; on the other hand, it can act as sponge and decoy by binding RBPs, preventing their specific cellular action such as regulation of cell cycle progression [236]. Furthermore, circRNAs have been reported to interact with mRNA. CircRNAs that contain translation start sites can act as mRNA traps and regulate protein translation of mRNA. Furthermore, several circRNAs have been reported to be capable of modulating the stability of mRNAs [237,238]. Finally, some circRNAs have the potential to be translated into proteins, a phenomenon first observed in the genome of RNA viruses [239]. Subsequent in vitro studies identified several protein-coding circRNAs in eukaryotes, and bioinformatic analyses of circRNA sequences have revealed the universal presence of circRNAs with coding potential in humans, leading to a new objective of functional circRNA studies [240,241]. Like miRNA and lncRNA, circRNA can be secreted from cells into the circulating bloodstream via extracellular vesicles such as exosomes, which are important carriers of these ncRNAs to distant target sites. Some exo-circRNAs have been shown to affect tumor cell proliferation, metastasis and therapy resistance [242].

In the clinical setting, liquid biopsy based on exo-circRNAs has been suggested to be a promising method for cancer diagnosis and prognosis, taking advantage of the fact that circRNAs are highly stable, enriched in exosomes and present in all body fluids. The expression profile of exo-circRNAs in patients with malignant tumors has been shown to differ from that in the healthy population [243]. Consequently, the search for exo-circRNAs suitable for cancer diagnosis and disease process monitoring is currently the focus of interest. In this regard, a recent Chinese study on a relatively small patient cohort has reported promising results. An exo-circRNA isolated from the plasma of gastric cancer patients was found to be closely associated with tumor size, TNM stage and lymph node metastasis. Furthermore, the expression level of this exo-circRNA decreased significantly after surgery, and the overall survival of patients with low expression of this exo-circRNA was reported to be longer than those with high expression. Thus, the authors suggested it to be a promising noninvasive biomarker for the diagnosis and prognosis of gastric cancer [244]. Further studies suggested different exo-circRNAs to have diagnostic and/or prognostic value for patients with, e.g., esophageal squamous cell carcinoma [245,246], BC [247,248], ovarian cancer [249] or hepatocellular carcinoma [250]. It can be expected that larger studies will follow to validate the relationship between the exo-circRNAs mentioned above and patients’ prognosis and to identify further exo-circRNAs or sets of these molecules associated with the survival of cancer patients. In addition to their putative role as prognostic biomarkers in cancer, the unique cellular stability and function of circRNAs in sponging miRNA and proteins may also indicate that exo-circRNAs are a promising vehicle for targeted therapy. In addition to the canonical, exonic circRNAs referred to above, which were the focus of the vast majority of studies, other classes of non-canonical circRNAs have been identified, namely lariat-derived intronic circRNAs, sub-exonic circRNAs, intron circles and tricRNAs [251]. Since these circRNA types are only beginning to be examined, they are only mentioned here for the sake of completeness but are not elaborated on in more detail.

#### CircRNAs Affecting Response to Endocrine Therapies by Mechanisms Involving Estrogen Signaling

An increasing number of circRNAs has been reported to affect the efficacy of endocrine therapies in BC patients by regulating different signaling pathways. A considerable part of these circRNAs was identified as modulating the expression of proteins acting as coregulators of estrogen signaling.

In a recent study, circRNA 0025202 was demonstrated to upregulate Forkhead box class O 3a (FOXO3a), a transcription factor and tumor suppressor associated with a positive response to tamoxifen therapy in Luminal A BC patients by sponging miR-182-5p [252]. The observed tamoxifen-sensitizing effect of circRNA 0025202 via the miR-182-5p/FOXO3a axis involves the activation of estrogen signaling, since FOXO3a is known to induce expression of *ESR1* [253]. Another circRNA, CDR1-AS, has been reported to sponge miR-7-5p, the high expression of which is predictive for a poor response to tamoxifen therapy [79,235]. Sponging of miR-7-5p by this circRNA leads to upregulation of the *FOS* gene, coding for a component of the AP-1 transcription factor, which is known to directly bind to ERα and to act as coactivator of this nuclear receptor [254]. These data strongly suggest that the circRNA CDR1-AS acts as a tamoxifen sensitizer via the miR-7-5p/FOS axis activating ERα signaling. A recent Chinese study reported that the circRNA circCDK1 sponges up miR-489-3p, leading to elevated CDK1 levels and a reduced tamoxifen response [255]. On the other hand, miR-489-3p is known to target and downregulate the *PTPN11* gene coding for tyrosine phosphatase SHP2, which activates the transcriptional activity of ERα, and this was shown to be essential for expression of estrogen target genes such as *CCND1* and *TFF1* [256,257]. Thus, circCDK1 is suggested to positively affect tamoxifen response via the circCDK1/miR-489-3p/SHP2 axis. Another very recent study suggested that circRNA TRIM28 enhances tamoxifen resistance via the miR-409-3p/HMGA2 axis. However, the potential mechanisms by which HMGA2 could affect tamoxifen response were not addressed, although a close relation of HMGA2 and ERα was identified in studies on cervical cancer and uterine leiomyoma [258,259,260]. In ERα-positive BC cells, the circRNA circTP63 was recently shown to sponge up miR-873-3p, thereby preventing the targeting and downregulation of *FOXM1* which, in turn, induces cell proliferation and invasion [261]. FOXM1 is known to regulate the transcriptional activity of ERα via interaction with the coactivator CARM1 and was reported to promote tamoxifen resistance in BC [64,179]. However, it remains to be investigated whether circTP63 is able to affect tamoxifen response via the miR-873-3p/FOXM1 axis. Finally, a recent study reported that circRNA circMET contributes to tamoxifen resistance of BC cells by sponging up miR-204, leading to sustained expression and activity of the aryl hydrocarbon receptor (AHR) [262]. Again, there is evidence that estrogen signaling is involved in this process, since AHR is well known to downregulate ERα signaling via different mechanisms such as proteasomal ERα degradation [263]. Recently, AHR has been reported to decrease the estrogen-triggered gene regulatory activity of ERα by formation of an AHR/ERα/RIP140 complex that reduces the activation of ERα response genes [264]. Suppressing estrogen signaling is known to result in tamoxifen resistance; however, the presumable negative effect of circMET on the tamoxifen response via miR-204/AHR/ERα remains to be validated. With regard to exosomal circRNAs, a recent study reported the ability of both in vitro and in vivo transfer of circRNA UBE2D2 by exosomes to confer tamoxifen resistance by sponging up miR-200a-3p [265]. Although in this study, the exact molecular mechanisms underlying this observation were not examined, miR-200a-3p is known to target and downregulate the *ZEB1* gene coding for the Zinc finger E-box-binding homeobox 1 protein, which has been demonstrated to repress *ESR1* transcription and to thereby induce tamoxifen resistance [266,267]. Thus, promotion of tamoxifen resistance by the circRNA UBED2 is suggested to be mediated by regulation of the miR-200-3p/ZEB1 axis.

Taken together, to date, most circRNAs affecting the response to endocrine therapies have been reported to affect tamoxifen sensitivity of BC cells by sponging up miRNAs targeting ERα coregulators or genes modulating *ESR1* expression.

### 2.4. Summary

An increasing amount of data suggests an important role of non-coding RNAs in the response of BC cells to ET drugs. This effect of ncRNAs acting on the tumor microenvironment and of circulating ncRNAs acting in an endocrine manner has been reported to be mediated by various cellular pathways. Here, we focused on ncRNAs modulating estrogen signaling, the pathway primarily targeted by endocrine therapies. In this regard, the vast majority of available studies, primarily at the in vitro level, examined the effect of ncRNAs on cellular tamoxifen sensitivity, whereas few studies reported an effect on the response to other SERMs, SERDs such as fulvestrant or aromatase inhibitors. A large number of miRNAs have been identified to target mRNAs of genes involved in estrogen signaling. Only a part of these studies also examined the effects of this interaction on the response to ET drugs; rather, most of them suggested that these miRNAs contribute to tamoxifen resistance. However, since the link to reduced estrogen signaling has become increasingly clear, it can be expected that future studies will clarify the role of all miRNAs targeting these genes in ET response of BC patients. In comparison to the vast miRNA data in existence, to date, significantly fewer studies exist that address the role of lncRNAs and circRNAs in this context. The lncRNA studies reviewed here show a variety of mechanisms to be involved in their effects on estrogen signaling and response to ET drugs. On the one hand, due to their well-known function as ceRNA sponging miRNAs, lncRNAs were reported either to sustain the expression of genes involved in estrogen signaling which contributes to tamoxifen sensitivity (lncRNAs ERLC1, DSCAM-AS1, LOL), or to promote AI sensitivity by sustaining CYP19A1 expression *(LINC00094*). On the other hand, other mechanisms of lncRNA action have been reported to promote endocrine resistance; these mechanisms include binding to proteins of the estrogen pathways (such as cyclin D1); preventing their degradation (lncRNA DILA); enhancing ERα chromatin occupancy (HOTAIR); activation of ERα leading to estrogen-independent signaling (HOTAIR), e.g., through the MAPK/ERK pathway (LINC-ROR); modulation of ERα splicing and expression (MALAT1); binding to ERα protecting it from fulvestrant-triggered degradation (H19); or coactivation of ERα enhancing the agonist activity of the tamoxifen-bound receptor (SRA1). Finally, only a limited number of studies have addressed the role of circRNAs in endocrine resistance. In some studies, the function of circRNAs as ceRNAs and miRNA sponges resulted in enhanced tamoxifen sensitivity, mediated by sustained expression of estrogen receptor (co-)activators such as FOXO3 or FOS, whereas other studies reported circRNAs sponging up miRNAs targeting mRNAs coding for ERα repressors such as AHR, leading to endocrine resistance.

**Figure 1:**
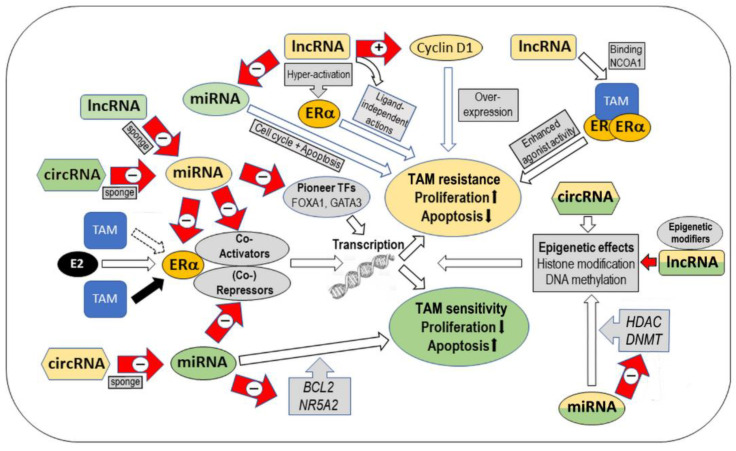
Schematic overview of mechanisms by which ncRNAs affect estrogen signaling and the response to tamoxifen (TAM) in BC cells. Red arrows indicate direct effects of non-coding RNAs. NcRNAs counteracting TAM resistance are colored in light green, ncRNAs in light orange color promote resistance to this SERM. More detailed information on the interactions shown here is provided in the text.

Taken together, the ncRNA network has been shown to affect estrogen signaling and the response to ET drugs in a positive or negative manner via highly complex interactions and a multitude of molecular mechanisms (Figure 1).

## 3. The Role of ncRNAs in Diagnosis, Prognosis and Prediction or Monitoring ET Response of BC Patients

Circulating ncRNAs have been shown to have particularly high potential as versatile, non-invasive biomarkers for cancer diagnosis, prognosis, and prediction of the therapy response.

### 3.1. NcRNAs and BC Diagnosis

Improvement of a BC patient’s outcome is directly related to early detection. Circulating ncRNAs are considered to be superior biomarkers for early BC diagnosis [268]. Here, a brief summary of exemplary studies is given, providing proof-of-concept. A number of reports showed various circulating cell-free (cf) miRNAs stabilized by exosomes or by AGO protein binding to exhibit a good diagnostic performance in the early detection of BC. A current comprehensive meta-analysis of such studies affirmed their diagnostic ability with a pooled sensitivity of 0.85 and a specificity of 0.83 and revealed a similar suitability of miRNAs isolated from plasma or serum. MiR-21-5p was the most analyzed miRNA in the included studies, showing a pooled sensitivity of 0.74 and a specificity of 0.81. However, subgroup analysis revealed that multiple miRNA panels had a better pooled diagnostic performance compared with single miRNA panels [269]. Circulating lncRNAs were also studied in this regard [270]. For example, circulating exosomal lncRNA H19 was reported to be elevated in BC patients compared with benign breast disease patients and healthy controls, and this was associated with lymph node metastasis, TNM stages and distant metastasis, suggesting that this lncRNA could be a novel biomarker for early BC diagnosis [271]. The role of circulating circRNAs in BC diagnosis has been addressed in a variety of recent studies [272]. For example, in a recent study, three tumor-derived circRNAs were identified and used to establish a diagnostic circRNA set with high combined specificity (0.98) and sensitivity (0.91) for early BC diagnosis [273].

### 3.2. NcRNAs and BC Prognosis

Circulating ncRNAs have also been shown to be a versatile tool for BC prognosis. A recent study analyzing total levels of circulating cf-miRNA demonstrated that high plasma levels of cf-miRNA were significantly associated with unfavorable clinical features, including tumor stage, tumor load and the presence of metastasis at diagnosis [274]. Various circulating miRNAs have been suggested to be potential biomarkers for BC prognosis [275]. Among them, levels of exosomal miR-148a were recently reported to be considerably lower in BC patients and low levels were associated with a poor clinical outcome in BC patients, suggesting this miRNA to be a potential prognostic biomarker [276]. Lymph node metastasis (LNM) represents an important independent risk factor for BC prognosis. Circulating exosomal miRNAs have been identified which were significantly associated with LNM, such as miR-363-5p and miR-148a, whereas others were correlated with bone or brain metastases [277]. Circulating lncRNAs and circRNAs have also been demonstrated to be potential biomarkers for BC prognosis [272,278]. However, further studies are needed to examine and validate the putative prognostic use of these ncRNA types for this cancer entity.

### 3.3. NcRNAs in Prediction and Monitoring Response to Endocrine Therapies

A multitude of ncRNAs have been shown either to enhance endocrine resistance in BC or to counteract it by affecting multiple signaling pathways. In this review article focusing on ncRNAs regulating estrogen signaling, the main target of ET, we have discussed a variety of miRNAs, lncRNAs and circRNAs that affect the expression of ERα or other key proteins involved in the cellular estrogen response. To date, only a part of these ncRNAs have been examined with regard to their effect on ET response. Thus, it can be expected that further studies will elucidate the role of these ncRNAs in endocrine resistance. However, it has to be considered that most of the data on this topic are based on results from vitro experiments, e.g., employing breast cancer cell lines. These are important because they allow to examine the molecular mechanisms of ncRNA action. However, more clinical studies correlating the expression of ncRNAs in BC patients with the ET response are necessary. With regard to the ncRNAs known to affect both estrogen signaling and endocrine response, their circulating forms, in particular, could serve as versatile biomarkers to predict or monitor the response to ET. In contrast, various studies analyzed intratumoral ncRNA expression in BC tissue and their implication for the ET response [279]. Today, analysis of circulating ncRNAs in liquid biopsies is considered to be a better and less invasive method [275,277], and initial studies exist that provide proof-of-concept [280]. Exosomal miR-221/222 were the first miRNAs shown to confer resistance to tamoxifen and fulvestrant in BC cells via downregulation of Erα [125,128]. In a recent study, plasma levels of miR-221 were reported to be higher in tamoxifen-treated patients with local recurrence and metastasis, and they were also found to be significantly correlated with tamoxifen resistance and to predict resistance to this SERM with a sensitivity of 82.35% and a specificity of 71.43%, suggesting this miRNA to be a marker for the tamoxifen response [281]. High plasma levels of circulating exosomal lncRNA HOTAIR have been reported to be associated with tamoxifen resistance, which might emerge from the known effects of HOTAIR on ERα activity [106,207,208,282]. However, the exact value of HOTAIR to determine the tamoxifen response remains to be examined. Taken together, as shown in this review article, a multitude of in vitro studies have identified ncRNAs capable of affecting estrogen signaling and the ET response. Now, more effort is needed to determine to what extent quantitative analysis of these ncRNAs, e.g., in liquid biopsies of BC patients, can be used to predict or monitor response to ET.

## 4. Prospects of ncRNA-Based Therapeutic Strategies to Improve ET Response

As shown in this review article, a variety of ncRNAs have been identified which either promote or counteract cellular resistance to ET drugs by modulation of estrogen signaling. From identification of these ncRNAs, potential new therapy options have emerged to improve the response to endocrine therapies. Generally, in this context, therapeutic approaches would be the possible either targeting and downregulating ncRNAs which decrease therapy response or delivering ncRNAs which enhance ET response. To target adverse miRNAs, different strategies can be used, such as the delivery of anti-miRNA oligonucleotides (antagomiRs) or the use of synthetic miRNA sponges. Additionally, various small molecule inhibitors have been identified which block the activity of specific miRNAs. Interestingly, miR-21, an miRNA targeting *ESR1* and promoting resistance to tamoxifen and fulvestrant, has been demonstrated to be inhibited by various small molecules at different levels, e.g., by blocking miR-21 transcription, binding to its pri-miRNA or pre-miRNA, inhibiting miR-21 export, interrupting DICER function disabling formation of a functional miRNA duplex or blocking miR-21 binding to its target mRNA [283]. On the other hand, synthetic mimetics of miRNAs promoting ET sensitivity, which are designed to act in a gene-specific manner, could be delivered using extracellular vehicles, liposomes, inorganic nanoparticles, polymer-based systems or by vehicle-free transfer. The proof-of-concept regarding these therapy strategies has been provided in a variety of in vitro and animal studies [284]. Therapeutic strategies based on lncRNAs modulating the ET response are another option to overcome endocrine resistance. LncRNAs promoting resistance can be targeted by specific siRNAs, antisense oligonucleotides (ASOs) or by modified lncRNAs exerting dominant-negative effects, thereby blocking the activity of the endogenous lncRNA [285,286,287]. Furthermore, the delivery of synthetic RNAs designed to provide a multitude of lncRNA binding sites might reduce the availability and function of a specific endogenous lncRNA. On the other hand, lncRNAs promoting ET sensitivity could be delivered into tumor cells by methods like the ones described for miRNA transfer. Finally, the high stability of circRNAs due to their lack of 5′-3′ polarity and a polyadenylated tail provides a considerable benefit for therapeutical strategies based on circRNA delivery. The function of circRNAs as molecular miRNA sponges could be used by delivery of synthetic circRNAs providing multiple binding sites for miRNAs promoting endocrine resistance. The additional functions of circRNAs, such as protein binding, regulation of gene expression and the protein-coding function of certain circRNAs, such as the exonic circRNAs (ecRNAs), provide further mechanisms which could be used in therapeutic approaches to overcome endocrine resistance [288].

However, given that ncRNA functions are highly complex and always have pleiotropic effects such as the regulation of multiple genes, which can include oncogenes or tumor suppressor genes, therapeutic targeting of ncRNAs or their delivery is considered to have a high risk of problematic side-effects. Thus, the design and delivery of ncRNA-based therapeutics has to be thoroughly optimized to avoid such side-effects and extensive in vitro and animal studies are necessary to demonstrate their safety. Thus, the clinical application of ncRNA-based therapies, which are expected to have the potential to significantly improve cancer therapy and to overcome therapy resistance, lies in the future. Until these safety concerns have been sufficiently addressed, ncRNAs can be clinically used as important biomarkers for diagnosis, prognosis and for prediction and monitoring of therapy response.

## 5. Conclusions

The ncRNA types addressed in this article—the miRNAs, lncRNAs and circRNAs—modulate estrogen signaling in BC cells by multiple molecular mechanisms, including highly complex interactions between ncRNAs, and can affect the sensitivity of BC cells to ET drugs in a positive or negative manner. To date, most of the data on this topic result from in vitro studies, primarily focusing on the cellular tamoxifen response, with miRNAs being the most extensively studied ncRNA type. However, initial studies analyzing the intratumoral expression of ncRNAs or, particularly, their circulating forms in the serum of BC patients demonstrated that several ncRNAs shown to affect both estrogen signaling and cellular response to ET drugs in vitro were also associated with ET sensitivity of BC patients. The vast number of in vitro studies identifying a multitude of ncRNAs affecting estrogen signaling and ET response is expected to increase efforts examining the correlation of these ncRNAs with ET response in BC patients, particularly quantifying their circulating forms, which have the potential to be versatile and non-invasive biomarkers to predict and monitor patients’ responses to specific ET regimens. Finally, ncRNA-based therapeutic strategies are expected to have high potential in cancer therapy, e.g., to overcome ET resistance in BC patients, but, considering the fact that ncRNAs generally regulate a set of different protein-coding genes, a high risk of unspecific actions resulting in problematic side-effects exists, which has to be thoroughly examined to ensure the safety of such therapeutics before their use in the clinical setting will be possible.
